# Synthesis, crystal structure and Hirshfeld surface analysis of 1,1′-[oxybis(ethane-2,1-di­yl)]bis­(2-methyl­sulfanyl-1*H*-benzo[*d*]imidazole)

**DOI:** 10.1107/S2056989025003809

**Published:** 2025-05-02

**Authors:** Ahmed Moussaif, Lhoussaine El Ghayati, Camille Kalonji Mubengayi, Abdulsalam Alsubari, El Mokhtar Essassi, Joel T. Mague, Youssef Ramli

**Affiliations:** a National Center for Nuclear Energy, Science and Technology,Rabat Morocco; b Laboratory of Heterocyclic Organic Chemistry Medicines Science Research Center Pharmacochemistry Competence Center Mohammed V University in Rabat Faculte des Sciences Av Ibn Battouta BP 1014 Rabat Morocco; c Laboratoire de Chimie et Biochimie, Institut Superieur des Techniques Medicales, Kinshasa, Republique Democratique, Congo; d Laboratory of Medicinal Chemistry, Faculty of Clinical Pharmacy, 21 September University, Yemen; e Department of Chemistry, Tulane University, New Orleans, LA 70118, USA; f Laboratory of Medicinal Chemistry Drug Sciences Research Center Faculty of Medicine and Pharmacy Mohammed V University in Rabat Morocco; Katholieke Universiteit Leuven, Belgium

**Keywords:** crystal structure, benzimidazole, thio­ether, hydrogen bond, π-stacking, C—H⋯π(ring) inter­actions

## Abstract

The asymmetric unit of the title compound, C_20_H_22_N_4_OS_2_, consists of two independent mol­ecules, one of which is disordered. In each mol­ecule, the mean planes of the terminal benzimidazole moieties are inclined to one another by about 68°. In the crystal, tetra­molecular strands are generated by C—H⋯N hydrogen bonds and C—H⋯π(ring) inter­actions and are linked by C—H⋯π(ring) and π-stacking inter­actions.

## Abstract 2

In the title compound, the terminal benzimidazole moieties are inclined to one another by about 68°. In the crystal, tetra­molecular strands are generated by C—H⋯N hydrogen bonds and C—H⋯π(ring) inter­actions and are linked by C—H⋯π(ring) and π-stacking inter­actions.

## 1. Chemical context

The benzimidazole ring system consists of a five-membered imidazole ring (with two nitro­gens included in the heterocyclic structure) fused to another aromatic ring. This structure gives benzimidazoles significant chemical stability and a range of biological properties (Obaid *et al.*, 2022 ▸), making them a subject of inter­est in the pharmacological field.

The benzimidazole ring is a well-known motif recognized for its chemical flexibility, allowing effective inter­action with various biological targets. As derivatives of this ring, bis­benzimidazoles share inter­esting physicochemical properties, such as their ability to inter­act with biological macromolecules like proteins, enzymes, and DNA. These inter­actions make them promising candidates for the development of drugs aimed at treating various diseases. Several bis­benzimidazole derivatives have been studied for their activity against various pathogens, including parasites and bacteria. Compounds from this class are used in the treatment of parasitic diseases such as giardiasis, amebiasis, and onchocerciasis. Additionally, some studies have revealed that bis­benzimidazoles possess anti­cancer properties. They work by inhibiting key enzymes in cancer cells, blocking cell division, or inducing mechanisms of programmed cell death (apoptosis). For instance, derivatives like levamisole, used in cancer treatments (Yadav *et al.*, 2018 ▸), have shown potential effects in stimulating the immune system and inhibiting tumor growth. Moreover, emerging research suggests that certain bis­benzimidazoles could be beneficial in the treatment of neurodegenerative diseases like Alzheimer’s disease (Algul *et al.*, 2025 ▸).

Continuing our research in this field (*e.g.* Missioui *et al.*, 2022 ▸), we synthesized the title compound 1,1′-[oxybis(ethane-2,1-di­yl)]bis­(2-methyl­sulfanyl-1*H*-benzo[*d*]imidazole) *via* an alkyl­ation reaction. We determined its mol­ecular and crystalline structures, and conducted a Hirshfeld surface analysis to analyze the inter­molecular inter­actions.
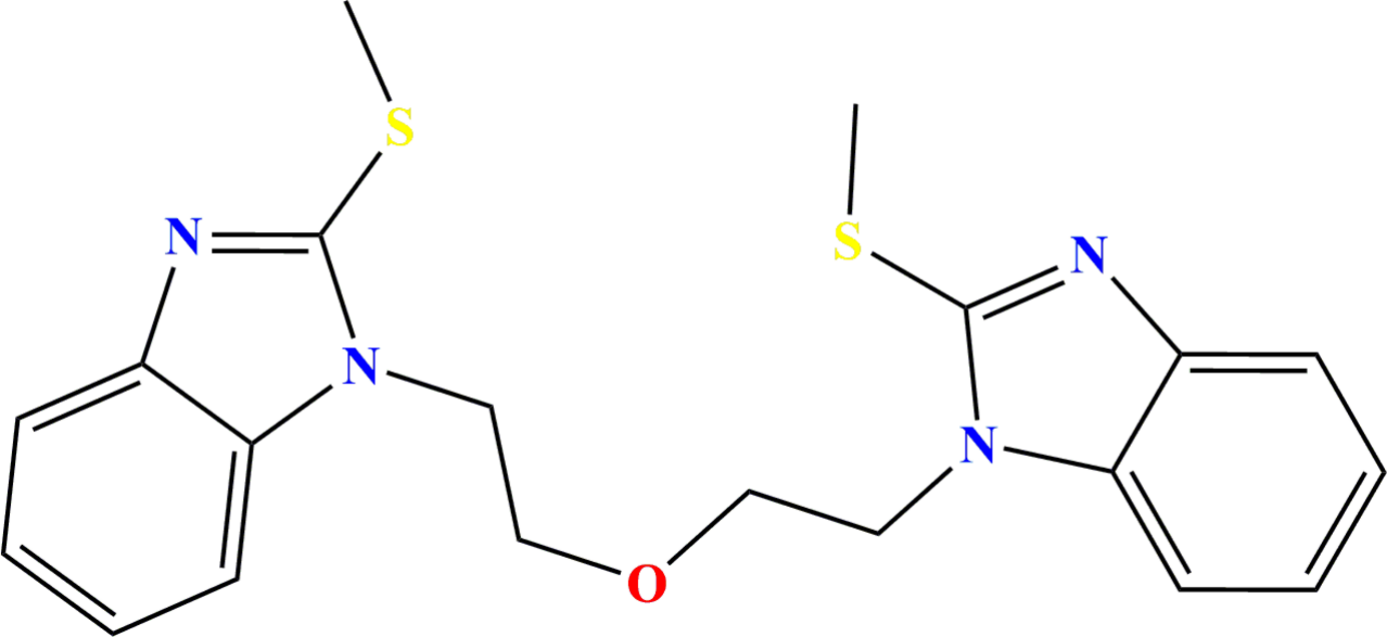


## 2. Structural commentary

**Figure 1:**
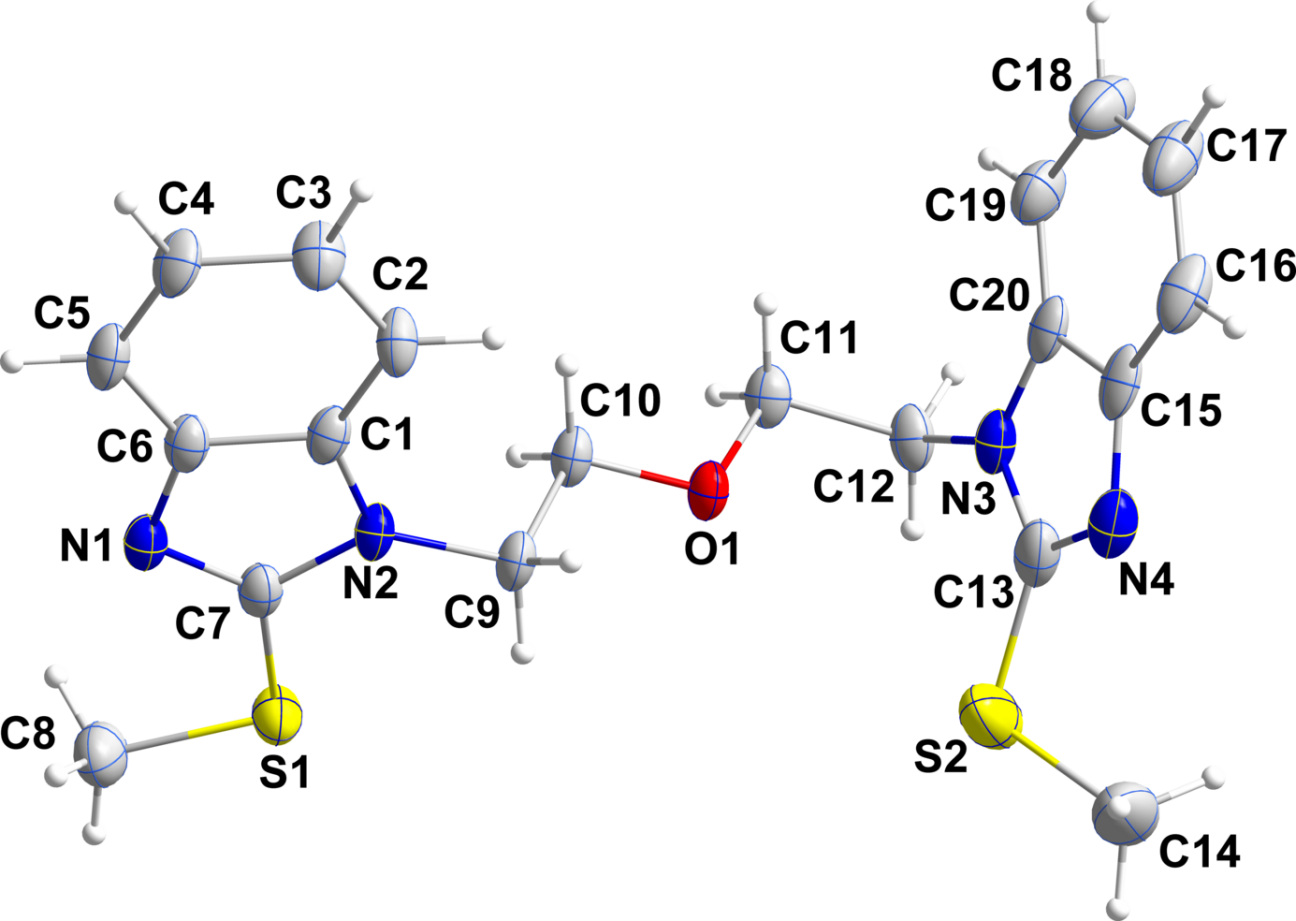
The ordered mol­ecule in the asymmetric unit of the title compound with labeling scheme and 50% probability ellipsoids.

**Figure 2:**
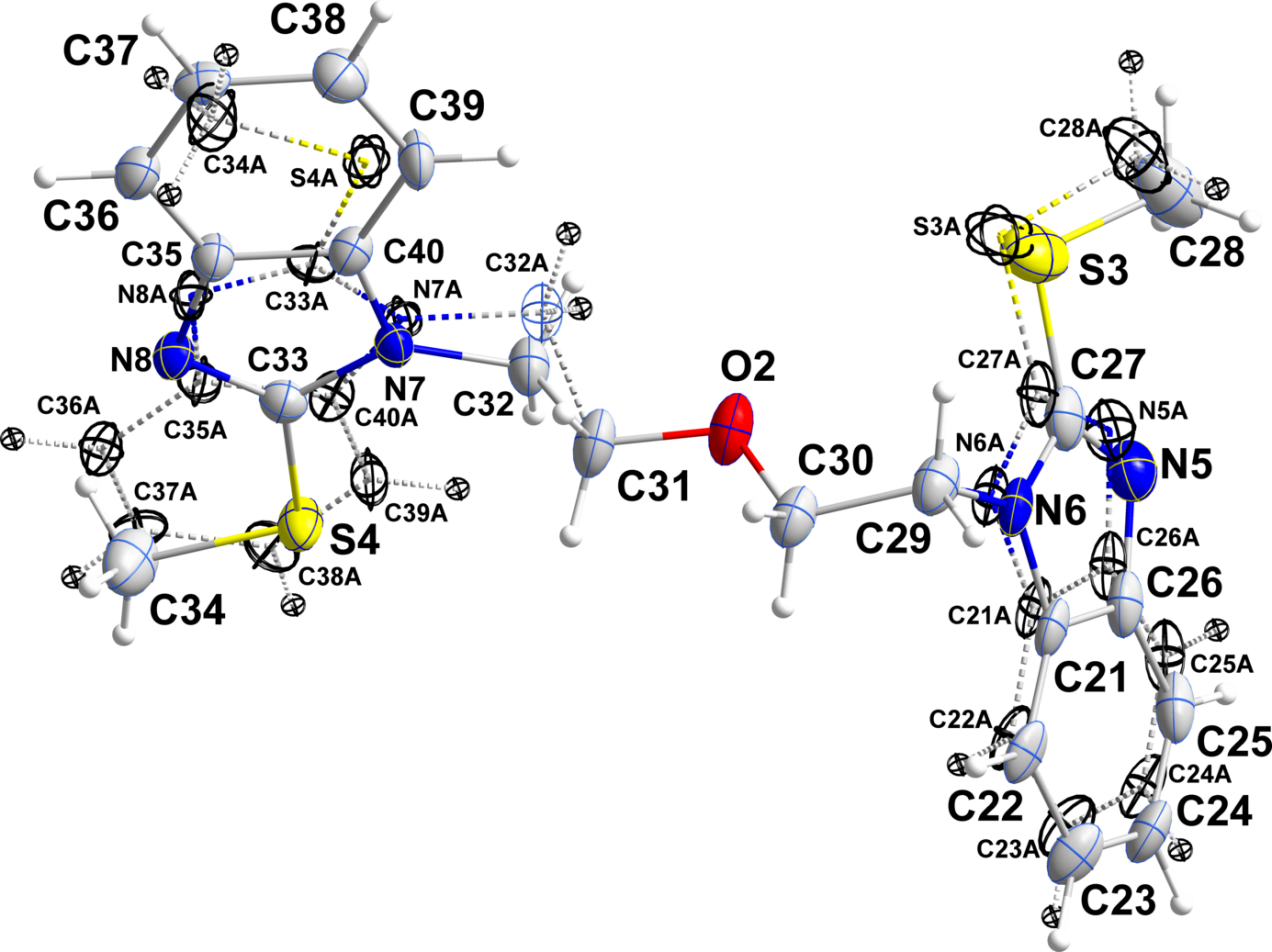
The disordered mol­ecule in the asymmetric unit of the title compound showing the overlay of the two components with the minor component depicted with dashed lines.

The asymmetric unit consists of two independent mol­ecules, one of which is disordered (Figs. 1 ▸ and 2 ▸). This involves the rotation of one 2-(methyl­sulfan­yl)benzimidazole unit by approximately 170° about the N7—C32 bond in a 0.7200 (13)/0.2800 (13) ratio, while for that at the other end of the mol­ecule a shift of 0.5 Å parallel to the plane of the unit is observed in a 0.775 (6)/0.225 (6) ratio (Fig. 2 ▸). In the ordered mol­ecule, the two benzimidazole units are nearly planar as the dihedral angles between their constituent planes are less than 2°, while the dihedral angle between the mean planes of the benzimidazole units in this mol­ecule is 68.38 (9)° (Fig. 1 ▸) and the C7—N2—C9 —C10 and the C13—N3—C12—C11 torsion angles are 99.1 (3) and 103.6 (3)°, respectively. In the central chain, the N2—C9—C10—O1 and the C11—O1—C10—C9 torsion angles are, respectively, −179.5 (2) and −180.0 (2)°, while the C10—O1—C11—C12 and the O1—C11—C12—N3 torsion angles are, respectively, 179.9 (2) and −60.0 (3)°. One methyl­sulfanyl group lies nearly in the plane of the five-membered ring to which it is attached [C8—S1—C7—N1 = −1.8 (3)°], but the other is rotated moderately out of the corresponding plane [C14—S2—C13—N4 = −23.2 (3)°]. The benzimidazole units in the disordered mol­ecule were refined as planar rigid groups and the dihedral angle between their mean planes in the major component is 68.12 (11)° but because of the disorder, comparison of its torsion angles with those of the ordered mol­ecule is not useful. In the ordered mol­ecule, bond distances and inter­bond angles are as expected for the formulation given.

## 3. Supra­molecular features

**Figure 3:**
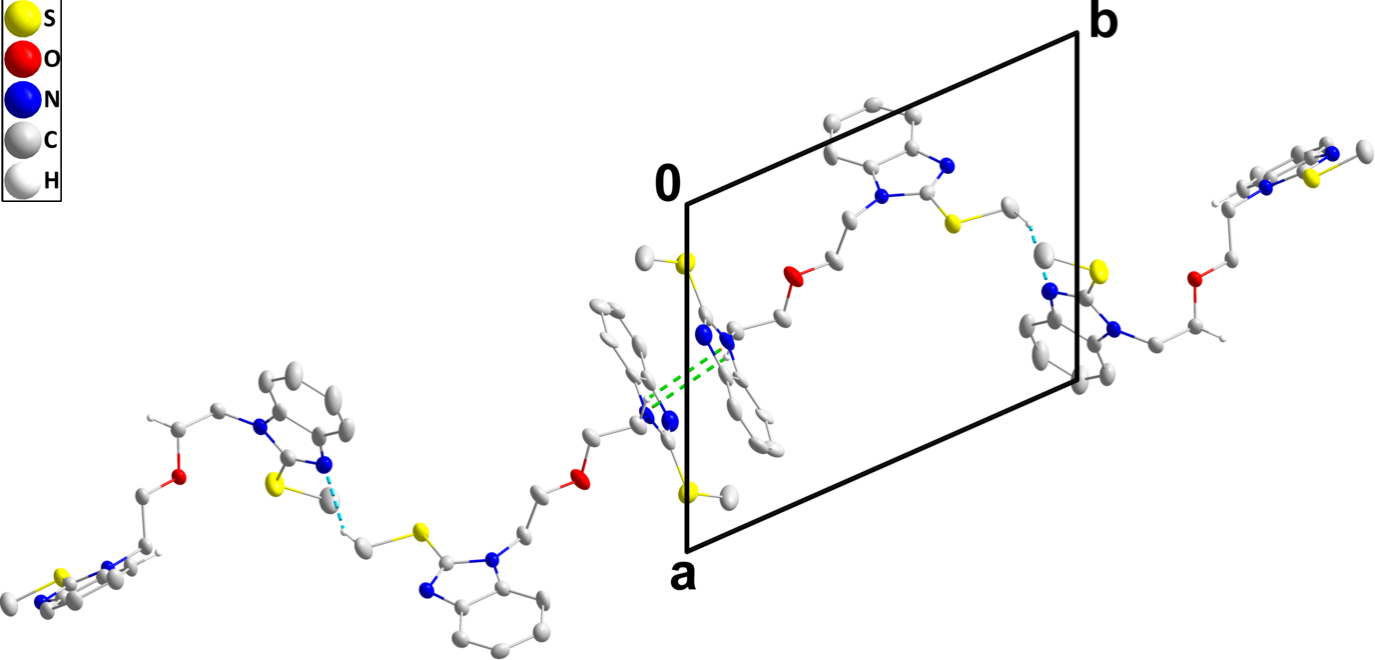
One tetra­molecular strand viewed along the *c*-axis direction with C—H⋯N hydrogen bonds and C—H⋯π(ring) inter­actions depicted, respectively, by blue and green dashed lines. Hydrogen atoms not involved in these inter­actions are omitted for clarity.

**Figure 4:**
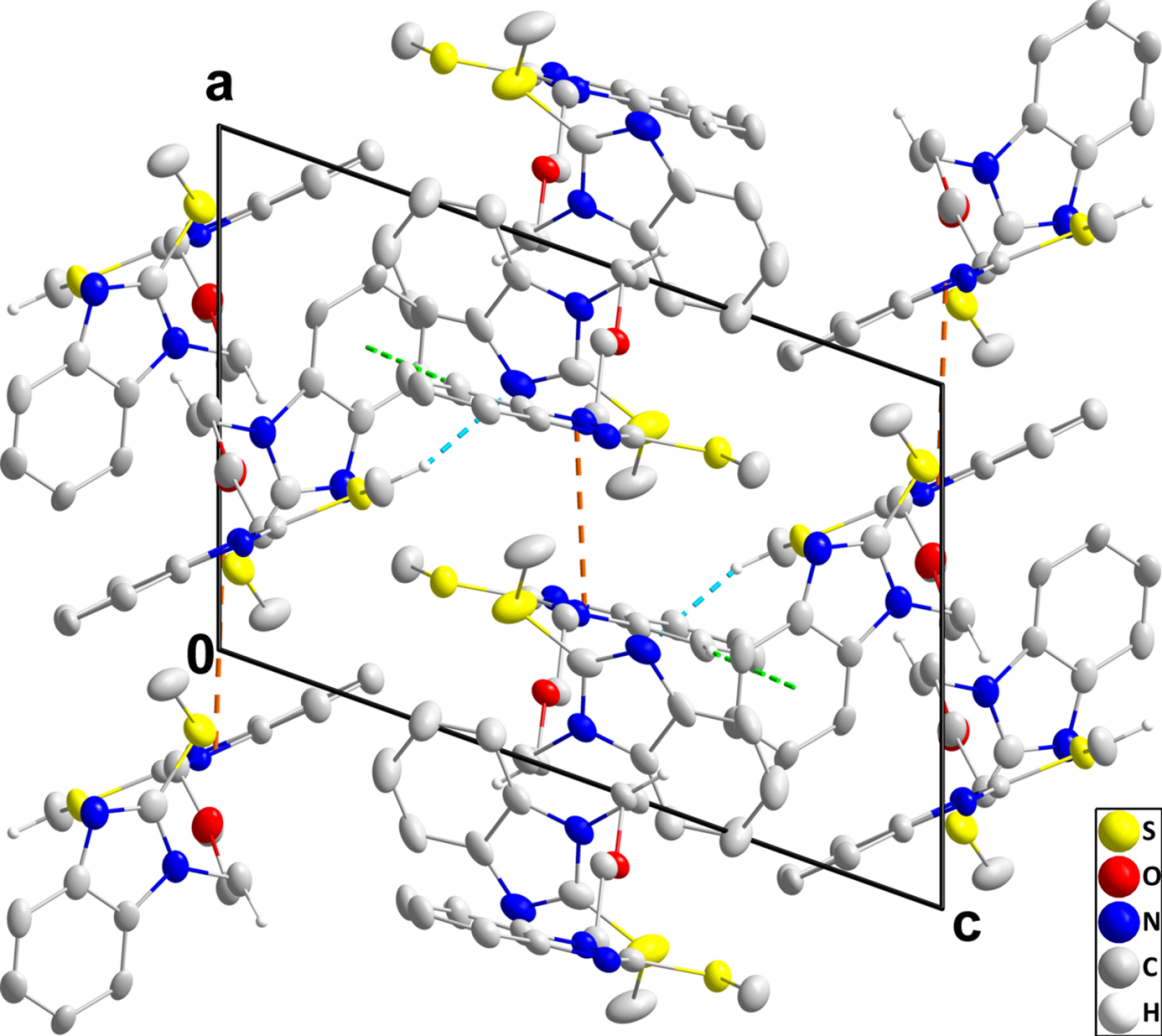
Packing viewed along the *b*-axis direction with C—H⋯N hydrogen bonds and C—H⋯π(ring) and π-stacking inter­actions depicted, respectively, by blue, green and orange dashed lines. Hydrogen atoms not involved in these inter­actions are omitted for clarity.

In the crystal, the major component of the disordered mol­ecule containing O2 is linked to the mol­ecule containing O1 at −*x* + 1, −*y* + 2, −*z* + 1 by a C35—H34*B*⋯N4 hydrogen bond (Table 1 ▸) and this two-mol­ecule unit is linked to its counterpart at −*x* + 1, −*y*, −*z* by a C29—H29*B*⋯*Cg*1 inter­action (Table 1 ▸ and Fig. 3 ▸). These tetra­molecular strands are connected by C2—H2⋯*Cg*5 and C11—H11B⋯*Cg*11 inter­actions (Table 1 ▸) as well as by π-stacking inter­actions between inversion-related N1/C6/C1/N2/C7 rings [centroid–centroid distance = 3.6645 (18) Å, dihedral angle = 0.03 (18)°, slippage = 1.06 Å] to generate the full 3-D structure (Fig. 4 ▸).

## 4. Database survey

**Figure 5:**
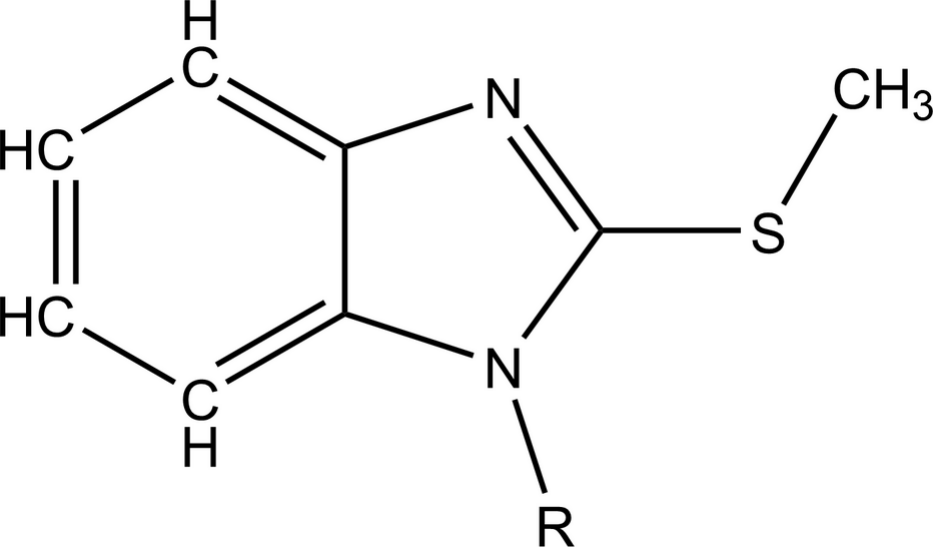
The fragment used for the database search.

A search of the Cambridge Structural Database (CSD updated to November 2024 (Groom *et al.*, 2016 ▸)) with the fragment shown in Fig. 5 ▸ (*R* = C) and restricted to only organic compounds generated seven hits. Four of these contained only one 2-(methyl­sulfan­yl)-1*H*-benzamidazole moiety and had *R* = CH_2_CH_2_OH (DUNZUI: Akonan *et al.*, 2010 ▸), 5,6-di­hydro-2*H*-pyran-2-one (IHAREP: Hammal *et al.*, 2008 ▸), morpholin-4-methyl (SIMCUN: Abou *et al.*, 2007 ▸) and the ionic compound 1-methyl-2-(methyl­sulfan­yl)-1*H*-benzimidazol-3-ium iodide (WANXUH: Hasty *et al.*, 2017 ▸). For these, the carbon atom of the methysulfanyl group lies in or very close to the plane of the benzimidazole moiety, while for the first three, the *R* group projects well out of that plane, which is similar to what is seen in the title mol­ecule. In the asymmetric unit of DUNZUI there are two independent mol­ecules and in its crystal packing, there are π-stacking inter­actions between five-membered rings of one of these. More extensive π-stacking occurs in WANXUH because of its relatively flat steric profile, while in SIMCUN both rings of the benzimidazole moiety participate in π-stacking inter­actions.

Two of the other examples are more analogous to the title mol­ecule with two 2-(methyl­sulfan­yl)-1*H*-benzamidazole moieties bridged by a —(CH_2_)_3_— chain (GEVJOH: Yüksektepe *et al.*, 2007 ▸) or by a 1,4-CH_2_C_6_H_4_CH_2_ unit (UGACEM: Rajakannu *et al.*, 2013 ▸) while the third has two 3-methyl-2-(methyl­sulfan­yl)-1*H*-benzimidazol-3-ium cations bridged by a 1,3-phenyl­ene group and triflate anions (KEYQUE: Steinke *et al.*, 2023 ▸). In GEVJOH, the dihedral angle between the mean planes of the two benzimidazole units is 74.87 (6)° while the torsion angles corresponding to the C7—N2—C9 —C10 and the C13—N3—C12—C11 torsion in the title mol­ecule are, 87.9 (2) and 93.6 (2)°, respectively, similar to the title compound. For the other two, the bridging units are much less flexible with the mean planes of the benzimidazole units in KEYQUE inclined to that of the central phenyl­ene ring by 60.5 (2) and 86.7 (2)°, respectively. UGACEM has crystallographically-imposed centrosymmetry and the unique benzimidazole is essentially perpendicular to the central phenyl­ene ring.

## 5. Hirshfeld surface analysis

**Figure 6:**
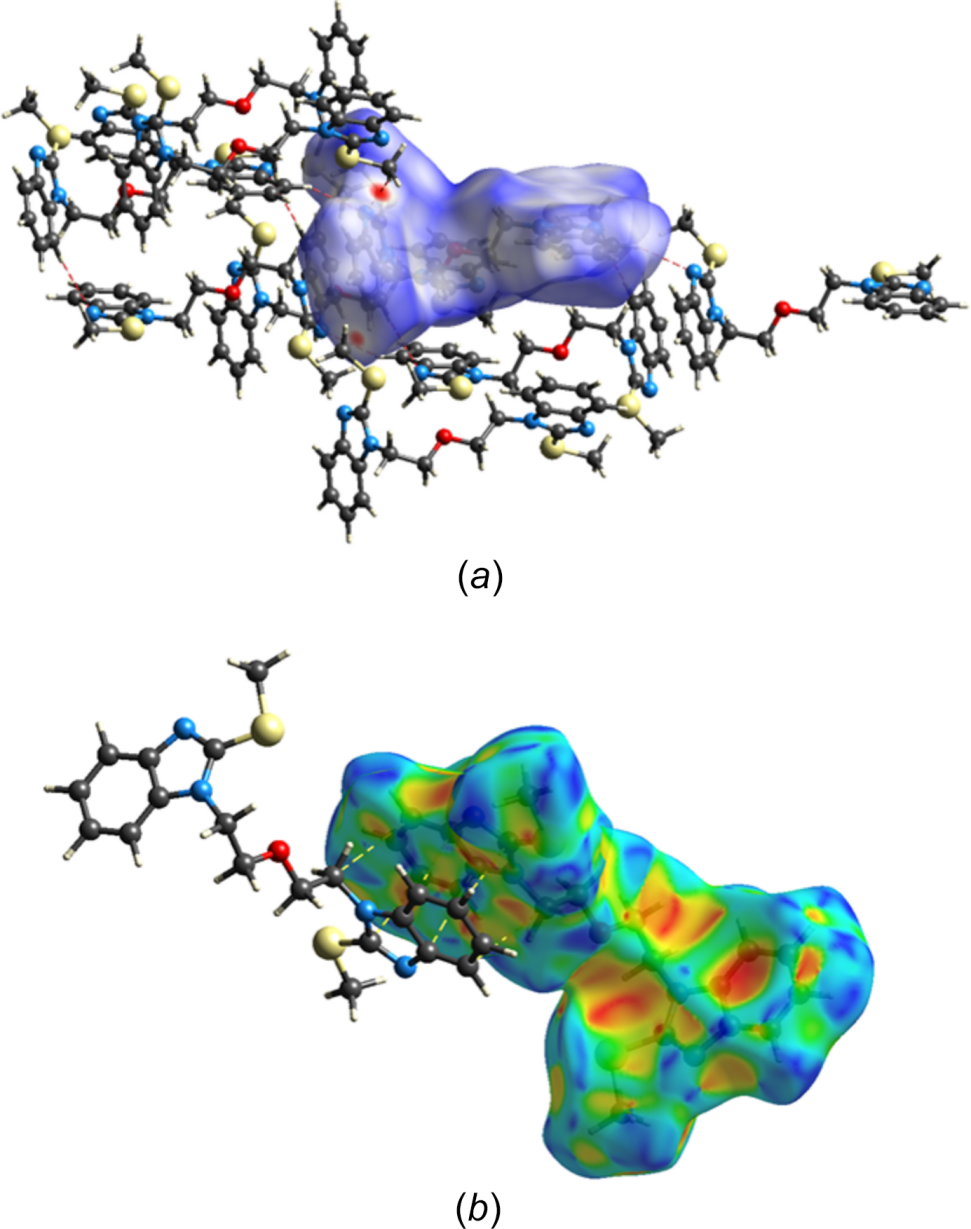
Hirshfeld surfaces: (*a*) the *d*_norm_ surface for the ordered mol­ecule with several nearest neighbors with C—H⋯N hydrogen bonds shown as dashed lines; (*b*) the surface calculated over the shape function for the major component of the disordered mol­ecule showing the π-stacking inter­action.

**Figure 7:**
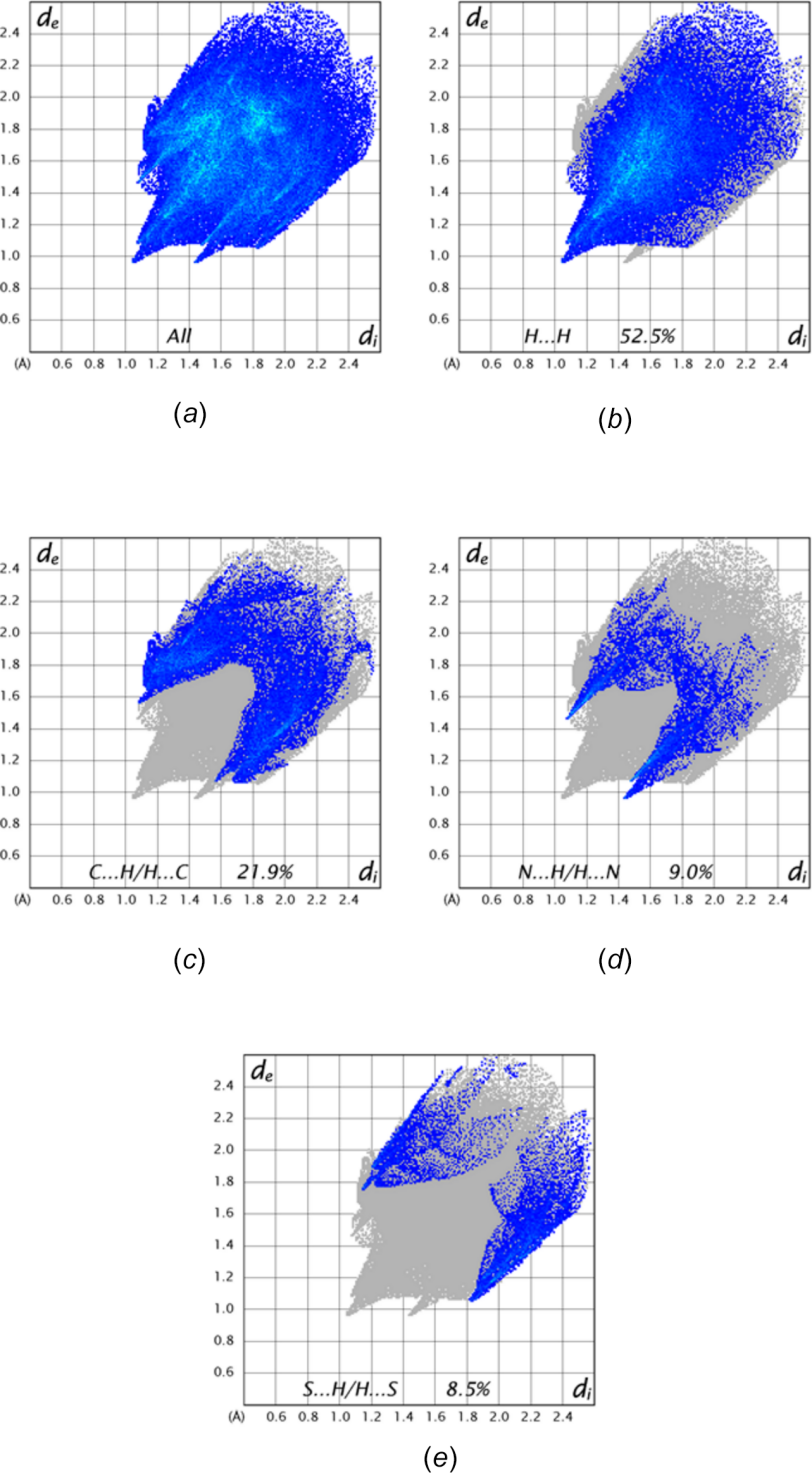
Fingerprint plots showing: (*a*) all inter­molecular inter­actions and those delineated into (*b*) H⋯H, (*c*) C⋯H/H⋯C, (*d*) N⋯H/H⋯N and (*e*) S⋯H/H⋯S contacts.

A Hirshfeld surface analysis of the title compound was performed with *CrystalExplorer* (Spackman *et al.*, 2021 ▸) to determine the contributions of the several inter­molecular inter­actions in the crystal. Full descriptions of the plots obtained and their inter­pretations have been published (Tan *et al.*, 2019 ▸). The *d*_norm_ surface for the mol­ecule containing O1 (ordered mol­ecule) calculated over the range −0.1811 to 1.3161 in arbitrary units together with several nearest neighbor mol­ecules including the major component of the disordered mol­ecule is shown in Fig. 6 ▸*a*. The C—H⋯N hydrogen bonds are depicted by red dashed lines and are clearly associated with the dark red spots on the *d*_norm_ surface. Fig. 6 ▸*b* shows the surface for the major component of the disordered mol­ecule calculated over the shape function and showing the characteristic pattern of triangles indicating the presence of the π-stacking inter­actions (dashed lines) noted in Section 3. The 2-D fingerprint plots for all inter­molecular inter­actions and those delineated into specific contacts are presented in Fig. 7 ▸. The largest contribution is from H⋯H contacts (Fig. 7 ▸*b*, 52.5% of the total) consistent with the significant hydrogen content of the mol­ecule and the fact that the hydrogen atoms constitute a large portion of its periphery. The next most important contact is C⋯H/H⋯C at 21.9% (Fig. 7 ▸*c*), which primarily comes from the C—H⋯π(ring) inter­actions. The N⋯H/H⋯N contacts (Fig. 7 ▸*d*), contributing 9.0%, appear as a pair of relatively sharp spikes at *d*_e_ + *d*_i_ = 2.88 Å and correspond primarily to the C—H⋯N hydrogen bonds while S⋯H/H⋯S contacts (Fig. 7 ▸*e*) contribute 8.5%. All other atom⋯atom contacts contribute a total of 8.1% and are considered quite minor.

## 6. Synthesis and crystallization

To a 50 mL round-bottom flask, 20 mL of di­methyl­formamide (DMF) were added followed by the successive addition of 0.0122 moles of 2-methyl­mercaptobenzimidazole, 0.0150 moles of potassium carbonate (K_2_CO_3_), 0.0070 moles of 1-chloro-2-(2-chloro­eth­oxy)ethane, and 0.0007 moles of tetra­butyl­ammonium bromide (BTBA). The mixture was stirred at room temperature for 2 h.

The salts were removed by filtration and the solvent was then removed under reduced pressure on a rotary evaporator. The residue obtained was subsequently purified by silica gel column chromatography, using hexa­ne/ethyl acetate (80/20, *v*/*v*) as the mobile phase and recrystallized from ethanol, yielding the title compound with a 72% yield as colorless crystals.

**^1^H NMR** (300 MHz, CDCl_3_) (δ, ppm): 2.54 (*s*, 6H), 3.73 (*t*, ^3^*J* = 7.5 Hz, 4H), 4.33 (*t*, ^3^J = 7.9 Hz, 4H), 7.48–7.82 (*m*, 8Har).; **^13^C NMR** (300 MHz, CDCl_3_) (δ, ppm): 15.5, 49.4, 71.3, 112.7, 124.4, 127.4, 138.8, 148.1, 171.2. **HRMS** (ESI–MS) (*m*/*z*) 398.54.

## 7. Refinement

Crystal data, data collection and structure refinement details are summarized in Table 2 ▸. H atoms attached to carbon were placed in calculated positions (C—H = 0.95–0.99 Å) and included as riding contributions with isotropic displacement parameters 1.2–1.5 times those of the attached atoms. In the mol­ecule containing O2, one end is disordered by a modest shift in a 0.775 (6)/0.225 (6) ratio while the other end is disordered by an approximate 170° rotation about the C31—C32 bond in a 0.7200 (13)/0.2800 (13) ratio. In both instances, the disordered portions were refined as rigid groups.

## Supplementary Material

Crystal structure: contains datablock(s) global, I. DOI: 10.1107/S2056989025003809/vm2313sup1.cif

Structure factors: contains datablock(s) I. DOI: 10.1107/S2056989025003809/vm2313Isup2.hkl

Supporting information file. DOI: 10.1107/S2056989025003809/vm2313Isup3.cml

CCDC reference: 2447131

Additional supporting information:  crystallographic information; 3D view; checkCIF report

## Figures and Tables

**Table 1:** Hydrogen-bond geometry (Å, °) *Cg*1, *Cg*5 and *Cg*11 are the centroids of the N5/C26/C21/N6/C27, the C21-C26 and the C1–C6 rings, respectively.

*D*—H⋯*A*	*D*—H	H⋯*A*	*D*⋯*A*	*D*—H⋯*A*
C2—H2⋯*Cg*5^i^	0.95	2.71	3.583 (3)	153
C11—H11*B*⋯*Cg*11^ii^	0.99	2.74	3.423 (3)	126
C29—H29*B*⋯*Cg*1^iii^	0.99	2.70	3.489 (3)	137
C34—H34*B*⋯N4^iv^	0.98	2.50	3.398 (3)	153

Symmetry codes: (i) 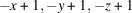; (ii) 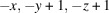; (iii) 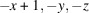; (iv) 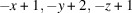.

**Table 2:** Experimental details

Crystal data	
Chemical formula	C_20_H_22_N_4_OS_2_
*M*_r_	398.53
Crystal system, space group	Triclinic, *P* 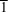
Temperature (K)	100
*a*, *b*, *c* (Å)	10.6654 (12), 13.5974 (15), 15.7917 (17)
α, β, γ (°)	109.206 (2), 101.687 (2), 107.789 (2)
*V* (Å^3^)	1938.8 (4)
*Z*	4
Radiation type	Mo *K*α
μ (mm^−1^)	0.29
Crystal size (mm)	0.36 × 0.22 × 0.18

Data collection	
Diffractometer	Bruker SMART APEX
Absorption correction	Multi-scan (*SADABS*; Krause *et al.*, 2015 ▸)
*T*_min_, *T*_max_	0.90, 0.95
No. of measured, independent and observed [*I* > 2σ(*I*)] reflections	36334, 10449, 5628
*R*_int_	0.074
(sin θ/λ)_max_ (Å^−1^)	0.693

Refinement	
*R*[*F*^2^ > 2σ(*F*^2^)], *wR*(*F*^2^), *S*	0.068, 0.199, 1.00
No. of reflections	10449
No. of parameters	460
No. of restraints	3
H-atom treatment	H-atom parameters constrained
Δρ_max_, Δρ_min_ (e Å^−3^)	1.32, −1.07

Computer programs: *APEX3* and *SAINT* (Bruker, 2015 ▸), *SHELXT* (Sheldrick, 2015*a* ▸), *SHELXL2019/1* (Sheldrick, 2015*b* ▸), *DIAMOND* (Brandenburg & Putz, 2012 ▸) and *SHELXTL* (Sheldrick, 2008 ▸).

## supplementary crystallographic information

### 1,1'-[Oxybis(ethane-2,1-diyl)]bis(2-methylsulfanyl-1H-benzo[d]imidazole) Crystal data

**Table:**

C_20_H_22_N_4_OS_2_	*Z* = 4
*M**_r_* = 398.53	*F*(000) = 840
Triclinic, *P*1	*D*_x_ = 1.365 Mg m^−^^3^
*a* = 10.6654 (12) Å	Mo *K*α radiation, λ = 0.71073 Å
*b* = 13.5974 (15) Å	Cell parameters from 9943 reflections
*c* = 15.7917 (17) Å	θ = 2.6–29.0°
α = 109.206 (2)°	µ = 0.29 mm^−^^1^
β = 101.687 (2)°	*T* = 100 K
γ = 107.789 (2)°	Column, colourless
*V* = 1938.8 (4) Å^3^	0.36 × 0.22 × 0.18 mm

### 1,1'-[Oxybis(ethane-2,1-diyl)]bis(2-methylsulfanyl-1H-benzo[d]imidazole) Data collection

**Table:**

Bruker SMART APEX diffractometer	10449 independent reflections
Radiation source: fine-focus sealed tube	5628 reflections with *I* > 2σ(*I*)
Graphite monochromator	*R*_int_ = 0.074
Detector resolution: 8.3333 pixels mm^-1^	θ_max_ = 29.5°, θ_min_ = 1.7°
φ and ω scans	*h* = −14→14
Absorption correction: multi-scan (*SADABS*; Krause *et al.*, 2015)	*k* = −18→18
*T*_min_ = 0.90, *T*_max_ = 0.95	*l* = −21→21
36334 measured reflections	

### 1,1'-[Oxybis(ethane-2,1-diyl)]bis(2-methylsulfanyl-1H-benzo[d]imidazole) Refinement

**Table:**

Refinement on *F*^2^	Primary atom site location: dual
Least-squares matrix: full	Secondary atom site location: difference Fourier map
*R*[*F*^2^ > 2σ(*F*^2^)] = 0.068	Hydrogen site location: inferred from neighbouring sites
*wR*(*F*^2^) = 0.199	H-atom parameters constrained
*S* = 1.00	*w* = 1/[σ^2^(*F*_o_^2^) + (0.1077*P*)^2^] where *P* = (*F*_o_^2^ + 2*F*_c_^2^)/3
10449 reflections	(Δ/σ)_max_ = 0.003
460 parameters	Δρ_max_ = 1.32 e Å^−^^3^
3 restraints	Δρ_min_ = −1.07 e Å^−^^3^

### 1,1'-[Oxybis(ethane-2,1-diyl)]bis(2-methylsulfanyl-1H-benzo[d]imidazole) Special details

**Table:**

Experimental. The diffraction data were obtained from 3 sets of 400 frames, each of width 0.5 deg. in omega, collected at phi = 0.00, 90.00 and 180.00 deg. and 2 sets of 800 frames, each of width 0.45 deg in phi, collected at omega = -30.00 and 210.00 deg. The scan time was 30 sec/frame.
Geometry. All esds (except the esd in the dihedral angle between two l.s. planes) are estimated using the full covariance matrix. The cell esds are taken into account individually in the estimation of esds in distances, angles and torsion angles; correlations between esds in cell parameters are only used when they are defined by crystal symmetry. An approximate (isotropic) treatment of cell esds is used for estimating esds involving l.s. planes.
Refinement. Refinement of F^2^ against ALL reflections. The weighted R-factor wR and goodness of fit S are based on F^2^, conventional R-factors R are based on F, with F set to zero for negative F^2^. The threshold expression of F^2^ > 2sigma(F^2^) is used only for calculating R-factors(gt) etc. and is not relevant to the choice of reflections for refinement. R-factors based on F^2^ are statistically about twice as large as those based on F, and R- factors based on ALL data will be even larger. H-atoms attached to carbon were placed in calculated positions (C—H = 0.95 - 0.99 Å). All were included as riding contributions with isotropic displacement parameters 1.2 - 1.5 times those of the attached atoms. In the molecule containing O2, one end is disordered by a modest shift in a 0.775 (6)/0.225 (6) ratio while the other end is disordered by an approximate 180° rotation about the C31—C32 bond in a 0.7200 (13)/0.2800 (13) ratio. In both instances, the disordered portions were refined as rigid groups.

### 1,1'-[Oxybis(ethane-2,1-diyl)]bis(2-methylsulfanyl-1H-benzo[d]imidazole) Fractional atomic coordinates and isotropic or equivalent isotropic displacement parameters (Å^2^)

**Table:**

	*x*	*y*	*z*	*U*_iso_*/*U*_eq_	Occ. (<1)
S1	0.28306 (8)	0.39883 (6)	0.31081 (5)	0.03271 (19)	
S2	0.28353 (10)	0.94354 (7)	0.40847 (8)	0.0589 (3)	
O1	0.14040 (17)	0.69787 (14)	0.45180 (12)	0.0265 (4)	
N1	0.3274 (2)	0.34561 (18)	0.46399 (16)	0.0261 (5)	
N2	0.3144 (2)	0.51604 (17)	0.49496 (15)	0.0245 (5)	
N3	0.0989 (2)	0.90589 (18)	0.50202 (16)	0.0287 (5)	
N4	0.2898 (2)	1.0698 (2)	0.58629 (18)	0.0370 (6)	
C1	0.3323 (3)	0.5043 (2)	0.57981 (18)	0.0254 (6)	
C2	0.3387 (3)	0.5740 (2)	0.66891 (19)	0.0301 (6)	
H2	0.332501	0.645270	0.681074	0.036*	
C3	0.3544 (3)	0.5346 (2)	0.73914 (19)	0.0347 (7)	
H3	0.358552	0.579523	0.801018	0.042*	
C4	0.3641 (3)	0.4298 (2)	0.7205 (2)	0.0331 (7)	
H4	0.374925	0.405259	0.770283	0.040*	
C5	0.3586 (3)	0.3602 (2)	0.6312 (2)	0.0306 (6)	
H5	0.365884	0.289325	0.619425	0.037*	
C6	0.3419 (3)	0.3990 (2)	0.56012 (19)	0.0256 (6)	
C7	0.3107 (3)	0.4180 (2)	0.42919 (19)	0.0253 (6)	
C8	0.2915 (4)	0.2620 (2)	0.2606 (2)	0.0437 (8)	
H8A	0.210903	0.203462	0.261097	0.066*	
H8B	0.378035	0.264102	0.298536	0.066*	
H8C	0.290012	0.244072	0.194921	0.066*	
C9	0.2979 (3)	0.6119 (2)	0.47908 (19)	0.0266 (6)	
H9A	0.319226	0.612552	0.421047	0.032*	
H9B	0.364700	0.684011	0.533683	0.032*	
C10	0.1505 (3)	0.6037 (2)	0.46773 (19)	0.0269 (6)	
H10A	0.082889	0.531822	0.413204	0.032*	
H10B	0.128798	0.604427	0.525953	0.032*	
C11	0.0038 (3)	0.6962 (2)	0.44035 (19)	0.0277 (6)	
H11A	−0.019845	0.697939	0.498150	0.033*	
H11B	−0.065672	0.625561	0.385448	0.033*	
C12	0.0002 (3)	0.7980 (2)	0.42393 (19)	0.0299 (6)	
H12A	0.021078	0.793568	0.364827	0.036*	
H12B	−0.095464	0.795720	0.414292	0.036*	
C13	0.2245 (3)	0.9775 (2)	0.5057 (2)	0.0360 (7)	
C14	0.3995 (4)	1.0841 (3)	0.4299 (3)	0.0683 (12)	
H14A	0.435052	1.079617	0.376729	0.103*	
H14B	0.478065	1.115441	0.489089	0.103*	
H14C	0.348430	1.133494	0.435353	0.103*	
C15	0.2020 (3)	1.0574 (2)	0.6399 (2)	0.0328 (7)	
C16	0.2183 (3)	1.1275 (2)	0.7317 (2)	0.0410 (8)	
H16	0.298217	1.197014	0.767967	0.049*	
C17	0.1160 (4)	1.0933 (3)	0.7681 (2)	0.0506 (9)	
H17	0.126520	1.139227	0.831367	0.061*	
C18	−0.0043 (4)	0.9924 (3)	0.7150 (2)	0.0517 (9)	
H18	−0.074229	0.972461	0.742407	0.062*	
C19	−0.0230 (3)	0.9218 (2)	0.6238 (2)	0.0384 (7)	
H19	−0.104208	0.853237	0.587287	0.046*	
C20	0.0820 (3)	0.9558 (2)	0.58821 (19)	0.0290 (6)	
O2	0.3432 (2)	0.27334 (17)	0.01613 (14)	0.0388 (5)	
S3	0.16784 (16)	−0.00367 (13)	0.02660 (19)	0.0477 (5)	0.775 (6)
N5	0.3973 (3)	0.04310 (18)	0.17227 (15)	0.0359 (8)	0.775 (6)
N6	0.44458 (15)	0.10270 (18)	0.06022 (10)	0.0285 (6)	0.775 (6)
C21	0.57282 (16)	0.13722 (15)	0.12777 (10)	0.0269 (7)	0.775 (6)
C22	0.70801 (15)	0.1992 (2)	0.13525 (16)	0.0274 (8)	0.775 (6)
H22	0.726802	0.224703	0.088098	0.033*	0.775 (6)
C23	0.81397 (19)	0.2219 (2)	0.21508 (18)	0.0331 (9)	0.775 (6)
H23	0.908138	0.264333	0.223266	0.040*	0.775 (6)
C24	0.7853 (3)	0.1837 (2)	0.28399 (14)	0.0332 (10)	0.775 (6)
H24	0.860720	0.200592	0.337720	0.040*	0.775 (6)
C25	0.6500 (3)	0.1219 (3)	0.27622 (13)	0.0349 (10)	0.775 (6)
H25	0.631661	0.096128	0.323322	0.042*	0.775 (6)
C26	0.5421 (2)	0.09900 (16)	0.19659 (11)	0.0315 (8)	0.775 (6)
C27	0.34510 (18)	0.04853 (11)	0.09217 (12)	0.0343 (8)	0.775 (6)
C28	0.1014 (6)	−0.1083 (4)	0.0703 (4)	0.0574 (16)	0.775 (6)
H28A	−0.000450	−0.147436	0.039998	0.086*	0.775 (6)
H28B	0.143558	−0.163816	0.055384	0.086*	0.775 (6)
H28C	0.124789	−0.070815	0.139505	0.086*	0.775 (6)
S3A	0.1493 (6)	0.0051 (5)	−0.0063 (5)	0.0477 (5)	0.225 (6)
N5A	0.3535 (8)	0.0386 (7)	0.1495 (5)	0.0359 (8)	0.225 (6)
N6A	0.4310 (6)	0.1156 (7)	0.0536 (4)	0.0285 (6)	0.225 (6)
C21A	0.5488 (6)	0.1448 (7)	0.1284 (4)	0.0269 (7)	0.225 (6)
C22A	0.6890 (6)	0.2104 (10)	0.1500 (6)	0.0274 (8)	0.225 (6)
H22A	0.720515	0.243208	0.109577	0.033*	0.225 (6)
C23A	0.7804 (7)	0.2254 (9)	0.2336 (6)	0.0331 (9)	0.225 (6)
H23A	0.877397	0.269824	0.251334	0.040*	0.225 (6)
C24A	0.7330 (9)	0.1763 (10)	0.2925 (5)	0.0332 (10)	0.225 (6)
H24A	0.798933	0.188189	0.349213	0.040*	0.225 (6)
C25A	0.5928 (10)	0.1110 (11)	0.2707 (5)	0.0349 (10)	0.225 (6)
H25A	0.561761	0.077973	0.311061	0.042*	0.225 (6)
C26A	0.4993 (8)	0.0958 (7)	0.1871 (4)	0.0315 (8)	0.225 (6)
C27A	0.3187 (6)	0.0537 (4)	0.0717 (4)	0.0343 (8)	0.225 (6)
C28A	0.067 (2)	−0.1135 (11)	0.0191 (15)	0.0574 (16)	0.225 (6)
H28D	−0.032413	−0.152168	−0.019878	0.086*	0.225 (6)
H28E	0.112093	−0.166856	0.004352	0.086*	0.225 (6)
H28F	0.076253	−0.086085	0.086683	0.086*	0.225 (6)
C29	0.4256 (3)	0.1246 (2)	−0.02772 (19)	0.0342 (7)	
H29A	0.329141	0.074043	−0.072048	0.041*	
H29B	0.490264	0.102472	−0.059150	0.041*	
C30	0.4495 (3)	0.2450 (2)	−0.0135 (2)	0.0330 (6)	
H30A	0.542073	0.298032	0.035349	0.040*	
H30B	0.448728	0.252538	−0.073792	0.040*	
C31	0.3493 (3)	0.3779 (2)	0.0136 (2)	0.0355 (7)	
H31A	0.322927	0.368331	−0.053359	0.043*	
H31B	0.446198	0.436242	0.047735	0.043*	
C32	0.2527 (5)	0.4171 (3)	0.0588 (3)	0.0296 (9)	0.7195 (12)
H32A	0.164360	0.351176	0.040756	0.036*	0.7195 (12)
H32B	0.296278	0.452162	0.128946	0.036*	0.7195 (12)
C32A	0.1981 (13)	0.3781 (6)	0.0241 (9)	0.0296 (9)	0.2805 (12)
H32C	0.119843	0.314492	−0.031603	0.036*	0.2805 (12)
H32D	0.188617	0.368449	0.082266	0.036*	0.2805 (12)
S4	0.39349 (9)	0.68180 (7)	0.19358 (6)	0.0311 (2)	0.7195 (12)
N7	0.22350 (13)	0.49926 (7)	0.02765 (7)	0.0211 (5)	0.7195 (12)
N8	0.21846 (13)	0.66717 (8)	0.03301 (7)	0.0223 (6)	0.7195 (12)
C33	0.27146 (8)	0.61603 (6)	0.07802 (5)	0.0231 (7)	0.7195 (12)
C34	0.41965 (18)	0.82874 (7)	0.22143 (9)	0.0458 (12)	0.7195 (12)
H34A	0.450478	0.850518	0.173404	0.069*	0.7195 (12)
H34B	0.491032	0.878050	0.284386	0.069*	0.7195 (12)
H34C	0.331546	0.836988	0.221551	0.069*	0.7195 (12)
C35	0.12425 (11)	0.57701 (9)	−0.05554 (6)	0.0244 (7)	0.7195 (12)
C36	0.03477 (17)	0.58012 (13)	−0.13155 (8)	0.0288 (8)	0.7195 (12)
H36	0.032523	0.649751	−0.130650	0.035*	0.7195 (12)
C37	−0.04984 (16)	0.47801 (15)	−0.20768 (7)	0.0264 (8)	0.7195 (12)
H37	−0.111558	0.477422	−0.260650	0.032*	0.7195 (12)
C38	−0.04811 (17)	0.37225 (13)	−0.20983 (8)	0.0354 (9)	0.7195 (12)
H38	−0.109548	0.303410	−0.263525	0.042*	0.7195 (12)
C39	0.04091 (19)	0.36846 (10)	−0.13557 (9)	0.0313 (9)	0.7195 (12)
H39	0.043377	0.298685	−0.137039	0.038*	0.7195 (12)
C40	0.12734 (12)	0.47218 (8)	−0.05802 (6)	0.0239 (7)	0.7195 (12)
S4A	0.0421 (3)	0.39830 (19)	−0.15751 (16)	0.0311 (2)	0.2805 (12)
N7A	0.1945 (4)	0.4857 (2)	0.02976 (17)	0.0211 (5)	0.2805 (12)
N8A	0.1597 (4)	0.61691 (18)	−0.02063 (18)	0.0223 (6)	0.2805 (12)
C33A	0.1366 (2)	0.50973 (18)	−0.04389 (15)	0.0231 (7)	0.2805 (12)
C34A	−0.0100 (5)	0.4769 (3)	−0.22092 (19)	0.0458 (12)	0.2805 (12)
H34D	0.073521	0.533118	−0.220876	0.069*	0.2805 (12)
H34E	−0.062280	0.515944	−0.189389	0.069*	0.2805 (12)
H34F	−0.069224	0.424149	−0.286721	0.069*	0.2805 (12)
C35A	0.2426 (3)	0.6713 (2)	0.07740 (18)	0.0244 (7)	0.2805 (12)
C36A	0.3022 (5)	0.7864 (2)	0.1396 (2)	0.0288 (8)	0.2805 (12)
H36A	0.287487	0.841980	0.119762	0.035*	0.2805 (12)
C37A	0.3831 (5)	0.8157 (3)	0.2307 (2)	0.0264 (8)	0.2805 (12)
H37A	0.424704	0.893413	0.274827	0.032*	0.2805 (12)
C38A	0.4068 (5)	0.7329 (4)	0.26154 (19)	0.0354 (9)	0.2805 (12)
H38A	0.464784	0.757051	0.324994	0.042*	0.2805 (12)
C39A	0.3472 (6)	0.6189 (3)	0.20094 (18)	0.0313 (9)	0.2805 (12)
H39A	0.361568	0.563534	0.221265	0.038*	0.2805 (12)
C40A	0.2647 (4)	0.5887 (2)	0.10817 (16)	0.0239 (7)	0.2805 (12)

### 1,1'-[Oxybis(ethane-2,1-diyl)]bis(2-methylsulfanyl-1H-benzo[d]imidazole) Atomic displacement parameters (Å^2^)

**Table:**

	*U*^11^	*U*^22^	*U*^33^	*U*^12^	*U*^13^	*U*^23^
S1	0.0413 (4)	0.0294 (4)	0.0308 (4)	0.0179 (3)	0.0115 (3)	0.0135 (3)
S2	0.0604 (6)	0.0366 (5)	0.0818 (7)	0.0194 (4)	0.0425 (5)	0.0165 (5)
O1	0.0267 (9)	0.0217 (9)	0.0318 (10)	0.0118 (8)	0.0049 (8)	0.0130 (8)
N1	0.0244 (11)	0.0220 (11)	0.0314 (12)	0.0103 (9)	0.0060 (9)	0.0117 (10)
N2	0.0258 (11)	0.0195 (11)	0.0274 (12)	0.0105 (9)	0.0036 (9)	0.0108 (9)
N3	0.0279 (12)	0.0205 (11)	0.0346 (13)	0.0108 (9)	0.0022 (10)	0.0118 (10)
N4	0.0293 (13)	0.0271 (13)	0.0509 (16)	0.0102 (10)	0.0052 (12)	0.0180 (12)
C1	0.0237 (13)	0.0214 (13)	0.0272 (14)	0.0074 (11)	0.0013 (11)	0.0117 (11)
C2	0.0361 (15)	0.0189 (13)	0.0272 (14)	0.0122 (11)	−0.0003 (12)	0.0056 (11)
C3	0.0446 (17)	0.0294 (15)	0.0226 (14)	0.0157 (13)	0.0009 (12)	0.0074 (12)
C4	0.0398 (16)	0.0295 (15)	0.0294 (15)	0.0150 (13)	0.0016 (12)	0.0167 (13)
C5	0.0317 (14)	0.0225 (13)	0.0345 (16)	0.0112 (11)	0.0023 (12)	0.0133 (12)
C6	0.0221 (13)	0.0219 (13)	0.0296 (14)	0.0083 (11)	0.0029 (11)	0.0109 (11)
C7	0.0225 (13)	0.0220 (13)	0.0306 (14)	0.0098 (11)	0.0064 (11)	0.0111 (11)
C8	0.060 (2)	0.0313 (16)	0.0424 (18)	0.0206 (15)	0.0217 (16)	0.0134 (15)
C9	0.0303 (14)	0.0197 (13)	0.0306 (14)	0.0112 (11)	0.0059 (11)	0.0130 (11)
C10	0.0294 (14)	0.0190 (12)	0.0308 (14)	0.0100 (11)	0.0050 (11)	0.0115 (11)
C11	0.0256 (13)	0.0232 (13)	0.0278 (14)	0.0094 (11)	−0.0004 (11)	0.0091 (11)
C12	0.0292 (14)	0.0244 (14)	0.0304 (15)	0.0134 (11)	0.0000 (11)	0.0085 (12)
C13	0.0309 (15)	0.0252 (15)	0.0519 (19)	0.0127 (12)	0.0090 (14)	0.0177 (14)
C14	0.070 (3)	0.043 (2)	0.107 (3)	0.0210 (19)	0.059 (3)	0.033 (2)
C15	0.0362 (15)	0.0224 (14)	0.0328 (15)	0.0101 (12)	−0.0059 (12)	0.0151 (12)
C16	0.0485 (19)	0.0257 (15)	0.0320 (16)	0.0055 (13)	−0.0047 (14)	0.0122 (13)
C17	0.080 (3)	0.0287 (16)	0.0340 (17)	0.0131 (17)	0.0156 (17)	0.0123 (14)
C18	0.072 (2)	0.0325 (17)	0.044 (2)	0.0095 (17)	0.0270 (18)	0.0151 (16)
C19	0.0450 (18)	0.0246 (15)	0.0377 (17)	0.0072 (13)	0.0098 (14)	0.0128 (13)
C20	0.0314 (14)	0.0215 (13)	0.0326 (15)	0.0105 (11)	−0.0001 (12)	0.0161 (12)
O2	0.0555 (13)	0.0383 (12)	0.0400 (12)	0.0330 (11)	0.0195 (10)	0.0219 (10)
S3	0.0413 (6)	0.0394 (6)	0.0431 (11)	0.0092 (5)	0.0076 (7)	0.0051 (7)
N5	0.044 (2)	0.0291 (14)	0.0327 (19)	0.0147 (16)	0.0109 (17)	0.0124 (14)
N6	0.0403 (14)	0.0231 (13)	0.0288 (13)	0.0213 (11)	0.0092 (11)	0.0122 (11)
C21	0.0387 (18)	0.0241 (14)	0.0267 (14)	0.0242 (14)	0.0103 (13)	0.0107 (12)
C22	0.0408 (18)	0.0338 (17)	0.0217 (16)	0.0308 (15)	0.0161 (13)	0.0103 (14)
C23	0.040 (2)	0.0467 (19)	0.0254 (19)	0.0306 (17)	0.0174 (15)	0.0143 (16)
C24	0.042 (3)	0.0400 (19)	0.0301 (17)	0.031 (2)	0.0099 (17)	0.0170 (15)
C25	0.060 (3)	0.0230 (17)	0.0246 (16)	0.023 (2)	0.0099 (19)	0.0104 (14)
C26	0.050 (3)	0.0226 (15)	0.0291 (17)	0.0231 (17)	0.0136 (17)	0.0116 (13)
C27	0.047 (2)	0.0216 (15)	0.037 (2)	0.0199 (15)	0.0151 (17)	0.0095 (15)
C28	0.060 (3)	0.036 (2)	0.071 (4)	0.014 (2)	0.041 (3)	0.011 (3)
S3A	0.0413 (6)	0.0394 (6)	0.0431 (11)	0.0092 (5)	0.0076 (7)	0.0051 (7)
N5A	0.044 (2)	0.0291 (14)	0.0327 (19)	0.0147 (16)	0.0109 (17)	0.0124 (14)
N6A	0.0403 (14)	0.0231 (13)	0.0288 (13)	0.0213 (11)	0.0092 (11)	0.0122 (11)
C21A	0.0387 (18)	0.0241 (14)	0.0267 (14)	0.0242 (14)	0.0103 (13)	0.0107 (12)
C22A	0.0408 (18)	0.0338 (17)	0.0217 (16)	0.0308 (15)	0.0161 (13)	0.0103 (14)
C23A	0.040 (2)	0.0467 (19)	0.0254 (19)	0.0306 (17)	0.0174 (15)	0.0143 (16)
C24A	0.042 (3)	0.0400 (19)	0.0301 (17)	0.031 (2)	0.0099 (17)	0.0170 (15)
C25A	0.060 (3)	0.0230 (17)	0.0246 (16)	0.023 (2)	0.0099 (19)	0.0104 (14)
C26A	0.050 (3)	0.0226 (15)	0.0291 (17)	0.0231 (17)	0.0136 (17)	0.0116 (13)
C27A	0.047 (2)	0.0216 (15)	0.037 (2)	0.0199 (15)	0.0151 (17)	0.0095 (15)
C28A	0.060 (3)	0.036 (2)	0.071 (4)	0.014 (2)	0.041 (3)	0.011 (3)
C29	0.0439 (17)	0.0308 (15)	0.0270 (15)	0.0225 (13)	0.0063 (13)	0.0076 (12)
C30	0.0437 (17)	0.0322 (15)	0.0267 (15)	0.0243 (13)	0.0080 (13)	0.0105 (12)
C31	0.0514 (18)	0.0339 (16)	0.0346 (16)	0.0293 (14)	0.0152 (14)	0.0186 (14)
C32	0.041 (3)	0.023 (2)	0.027 (3)	0.016 (2)	0.0091 (19)	0.0118 (19)
C32A	0.041 (3)	0.023 (2)	0.027 (3)	0.016 (2)	0.0091 (19)	0.0118 (19)
S4	0.0366 (5)	0.0262 (5)	0.0259 (5)	0.0137 (4)	0.0005 (4)	0.0104 (4)
N7	0.0230 (14)	0.0191 (12)	0.0247 (12)	0.0087 (10)	0.0093 (10)	0.0120 (10)
N8	0.0272 (17)	0.0227 (16)	0.0187 (16)	0.0118 (13)	0.0055 (12)	0.0102 (13)
C33	0.0222 (17)	0.0232 (17)	0.0217 (17)	0.0077 (14)	0.0074 (13)	0.0083 (14)
C34	0.054 (3)	0.033 (2)	0.036 (2)	0.024 (2)	0.003 (2)	0.0001 (19)
C35	0.0278 (18)	0.0218 (17)	0.0227 (19)	0.0111 (15)	0.0054 (15)	0.0092 (15)
C36	0.0316 (19)	0.0306 (19)	0.0287 (19)	0.0161 (16)	0.0084 (15)	0.0154 (16)
C37	0.0229 (17)	0.035 (2)	0.0168 (17)	0.0100 (15)	0.0023 (14)	0.0100 (16)
C38	0.036 (2)	0.031 (2)	0.029 (2)	0.0118 (17)	0.0061 (17)	0.0058 (17)
C39	0.043 (2)	0.0206 (18)	0.033 (2)	0.0149 (17)	0.0077 (17)	0.0149 (16)
C40	0.0272 (18)	0.0255 (18)	0.0216 (17)	0.0146 (15)	0.0075 (14)	0.0099 (14)
S4A	0.0366 (5)	0.0262 (5)	0.0259 (5)	0.0137 (4)	0.0005 (4)	0.0104 (4)
N7A	0.0230 (14)	0.0191 (12)	0.0247 (12)	0.0087 (10)	0.0093 (10)	0.0120 (10)
N8A	0.0272 (17)	0.0227 (16)	0.0187 (16)	0.0118 (13)	0.0055 (12)	0.0102 (13)
C33A	0.0222 (17)	0.0232 (17)	0.0217 (17)	0.0077 (14)	0.0074 (13)	0.0083 (14)
C34A	0.054 (3)	0.033 (2)	0.036 (2)	0.024 (2)	0.003 (2)	0.0001 (19)
C35A	0.0278 (18)	0.0218 (17)	0.0227 (19)	0.0111 (15)	0.0054 (15)	0.0092 (15)
C36A	0.0316 (19)	0.0306 (19)	0.0287 (19)	0.0161 (16)	0.0084 (15)	0.0154 (16)
C37A	0.0229 (17)	0.035 (2)	0.0168 (17)	0.0100 (15)	0.0023 (14)	0.0100 (16)
C38A	0.036 (2)	0.031 (2)	0.029 (2)	0.0118 (17)	0.0061 (17)	0.0058 (17)
C39A	0.043 (2)	0.0206 (18)	0.033 (2)	0.0149 (17)	0.0077 (17)	0.0149 (16)
C40A	0.0272 (18)	0.0255 (18)	0.0216 (17)	0.0146 (15)	0.0075 (14)	0.0099 (14)

### 1,1'-[Oxybis(ethane-2,1-diyl)]bis(2-methylsulfanyl-1H-benzo[d]imidazole) Geometric parameters (Å, º)

**Table:**

S1—C7	1.749 (3)	S3A—C27A	1.7474
S1—C8	1.805 (3)	S3A—C28A	1.799 (5)
S2—C13	1.753 (3)	N5A—C27A	1.3103
S2—C14	1.805 (3)	N5A—C26A	1.3998
O1—C10	1.414 (3)	N6A—C29	1.321 (6)
O1—C11	1.423 (3)	N6A—C27A	1.3808
N1—C7	1.314 (3)	N6A—C21A	1.3843
N1—C6	1.402 (3)	C21A—C22A	1.3879
N2—C7	1.377 (3)	C21A—C26A	1.4038
N2—C1	1.383 (3)	C22A—C23A	1.3840
N2—C9	1.460 (3)	C22A—H22A	0.9500
N3—C13	1.371 (4)	C23A—C24A	1.3994
N3—C20	1.383 (4)	C23A—H23A	0.9500
N3—C12	1.457 (3)	C24A—C25A	1.3863
N4—C13	1.317 (4)	C24A—H24A	0.9500
N4—C15	1.393 (4)	C25A—C26A	1.3929
C1—C2	1.388 (4)	C25A—H25A	0.9500
C1—C6	1.402 (3)	C28A—H28D	0.9800
C2—C3	1.383 (4)	C28A—H28E	0.9800
C2—H2	0.9500	C28A—H28F	0.9800
C3—C4	1.399 (4)	C29—C30	1.508 (4)
C3—H3	0.9500	C29—H29A	0.9900
C4—C5	1.392 (4)	C29—H29B	0.9900
C4—H4	0.9500	C30—H30A	0.9900
C5—C6	1.392 (4)	C30—H30B	0.9900
C5—H5	0.9500	C31—C32	1.495 (5)
C8—H8A	0.9800	C31—C32A	1.654 (13)
C8—H8B	0.9800	C31—H31A	0.9900
C8—H8C	0.9800	C31—H31B	0.9900
C9—C10	1.511 (4)	C32—N7	1.449 (3)
C9—H9A	0.9900	C32—H32A	0.9900
C9—H9B	0.9900	C32—H32B	0.9900
C10—H10A	0.9900	C32A—N7A	1.449 (4)
C10—H10B	0.9900	C32A—H32C	0.9900
C11—C12	1.500 (3)	C32A—H32D	0.9900
C11—H11A	0.9900	S4—C33	1.7540
C11—H11B	0.9900	S4—C34	1.8152
C12—H12A	0.9900	N7—C40	1.3773
C12—H12B	0.9900	N7—C33	1.3868
C14—H14A	0.9800	N8—C33	1.3081
C14—H14B	0.9800	N8—C35	1.4206
C14—H14C	0.9800	C34—H34A	0.9800
C15—C16	1.388 (4)	C34—H34B	0.9800
C15—C20	1.402 (4)	C34—H34C	0.9800
C16—C17	1.363 (5)	C35—C36	1.3954
C16—H16	0.9500	C35—C40	1.4237
C17—C18	1.401 (4)	C36—C37	1.3726
C17—H17	0.9500	C36—H36	0.9500
C18—C19	1.375 (4)	C37—C38	1.4330
C18—H18	0.9500	C37—H37	0.9500
C19—C20	1.376 (4)	C38—C39	1.3777
C19—H19	0.9500	C38—H38	0.9500
O2—C31	1.418 (3)	C39—C40	1.3966
O2—C30	1.420 (3)	C39—H39	0.9500
S3—C27	1.7474	S4A—C33A	1.7540
S3—C28	1.798 (5)	S4A—C34A	1.8152
N5—C27	1.3103	N7A—C40A	1.3773
N5—C26	1.3998	N7A—C33A	1.3867
N6—C27	1.3808	N8A—C33A	1.3081
N6—C21	1.3843	N8A—C35A	1.4206
N6—C29	1.501 (3)	C34A—H34D	0.9800
C21—C22	1.3879	C34A—H34E	0.9800
C21—C26	1.4038	C34A—H34F	0.9800
C22—C23	1.3839	C35A—C36A	1.3954
C22—H22	0.9500	C35A—C40A	1.4237
C23—C24	1.3994	C36A—C37A	1.3726
C23—H23	0.9500	C36A—H36A	0.9500
C24—C25	1.3863	C37A—C38A	1.4330
C24—H24	0.9500	C37A—H37A	0.9500
C25—C26	1.3930	C38A—C39A	1.3776
C25—H25	0.9500	C38A—H38A	0.9500
C28—H28A	0.9800	C39A—C40A	1.3966
C28—H28B	0.9800	C39A—H39A	0.9500
C28—H28C	0.9800		
			
C7—S1—C8	100.14 (14)	C29—N6A—C21A	127.8 (3)
C13—S2—C14	99.88 (16)	C27A—N6A—C21A	106.1
C10—O1—C11	111.49 (18)	N6A—C21A—C22A	131.6
C7—N1—C6	103.9 (2)	N6A—C21A—C26A	105.4
C7—N2—C1	106.1 (2)	C22A—C21A—C26A	123.0
C7—N2—C9	127.7 (2)	C23A—C22A—C21A	116.4
C1—N2—C9	126.2 (2)	C23A—C22A—H22A	121.8
C13—N3—C20	106.6 (2)	C21A—C22A—H22A	121.8
C13—N3—C12	127.9 (3)	C22A—C23A—C24A	121.4
C20—N3—C12	125.5 (2)	C22A—C23A—H23A	119.3
C13—N4—C15	104.2 (2)	C24A—C23A—H23A	119.3
N2—C1—C2	131.7 (2)	C25A—C24A—C23A	122.0
N2—C1—C6	105.5 (2)	C25A—C24A—H24A	119.0
C2—C1—C6	122.7 (2)	C23A—C24A—H24A	119.0
C3—C2—C1	116.8 (2)	C24A—C25A—C26A	117.4
C3—C2—H2	121.6	C24A—C25A—H25A	121.3
C1—C2—H2	121.6	C26A—C25A—H25A	121.3
C2—C3—C4	121.0 (3)	C25A—C26A—N5A	129.7
C2—C3—H3	119.5	C25A—C26A—C21A	119.9
C4—C3—H3	119.5	N5A—C26A—C21A	110.3
C5—C4—C3	122.1 (3)	N5A—C27A—N6A	114.0
C5—C4—H4	118.9	N5A—C27A—S3A	126.5
C3—C4—H4	118.9	N6A—C27A—S3A	119.5
C6—C5—C4	117.1 (2)	S3A—C28A—H28D	109.5
C6—C5—H5	121.5	S3A—C28A—H28E	109.5
C4—C5—H5	121.5	H28D—C28A—H28E	109.5
C5—C6—C1	120.2 (2)	S3A—C28A—H28F	109.5
C5—C6—N1	129.4 (2)	H28D—C28A—H28F	109.5
C1—C6—N1	110.3 (2)	H28E—C28A—H28F	109.5
N1—C7—N2	114.2 (2)	N6A—C29—C30	109.5 (4)
N1—C7—S1	126.1 (2)	N6—C29—C30	116.4 (2)
N2—C7—S1	119.69 (19)	N6—C29—H29A	108.2
S1—C8—H8A	109.5	C30—C29—H29A	108.2
S1—C8—H8B	109.5	N6—C29—H29B	108.2
H8A—C8—H8B	109.5	C30—C29—H29B	108.2
S1—C8—H8C	109.5	H29A—C29—H29B	107.3
H8A—C8—H8C	109.5	O2—C30—C29	110.0 (2)
H8B—C8—H8C	109.5	O2—C30—H30A	109.7
N2—C9—C10	110.6 (2)	C29—C30—H30A	109.7
N2—C9—H9A	109.5	O2—C30—H30B	109.7
C10—C9—H9A	109.5	C29—C30—H30B	109.7
N2—C9—H9B	109.5	H30A—C30—H30B	108.2
C10—C9—H9B	109.5	O2—C31—C32	110.8 (2)
H9A—C9—H9B	108.1	O2—C31—C32A	102.3 (3)
O1—C10—C9	108.1 (2)	O2—C31—H31A	109.5
O1—C10—H10A	110.1	C32—C31—H31A	109.5
C9—C10—H10A	110.1	O2—C31—H31B	109.5
O1—C10—H10B	110.1	C32—C31—H31B	109.5
C9—C10—H10B	110.1	H31A—C31—H31B	108.1
H10A—C10—H10B	108.4	N7—C32—C31	109.8 (3)
O1—C11—C12	108.8 (2)	N7—C32—H32A	109.7
O1—C11—H11A	109.9	C31—C32—H32A	109.7
C12—C11—H11A	109.9	N7—C32—H32B	109.7
O1—C11—H11B	109.9	C31—C32—H32B	109.7
C12—C11—H11B	109.9	H32A—C32—H32B	108.2
H11A—C11—H11B	108.3	N7A—C32A—C31	108.3 (7)
N3—C12—C11	113.5 (2)	N7A—C32A—H32C	110.0
N3—C12—H12A	108.9	C31—C32A—H32C	110.0
C11—C12—H12A	108.9	N7A—C32A—H32D	110.0
N3—C12—H12B	108.9	C31—C32A—H32D	110.0
C11—C12—H12B	108.9	H32C—C32A—H32D	108.4
H12A—C12—H12B	107.7	C33—S4—C34	99.7
N4—C13—N3	113.6 (3)	C40—N7—C33	105.9
N4—C13—S2	126.0 (2)	C40—N7—C32	124.77 (17)
N3—C13—S2	120.4 (2)	C33—N7—C32	129.05 (17)
S2—C14—H14A	109.5	C33—N8—C35	103.7
S2—C14—H14B	109.5	N8—C33—N7	115.1
H14A—C14—H14B	109.5	N8—C33—S4	125.9
S2—C14—H14C	109.5	N7—C33—S4	119.0
H14A—C14—H14C	109.5	S4—C34—H34A	109.5
H14B—C14—H14C	109.5	S4—C34—H34B	109.5
C16—C15—N4	130.1 (3)	H34A—C34—H34B	109.5
C16—C15—C20	119.5 (3)	S4—C34—H34C	109.5
N4—C15—C20	110.5 (3)	H34A—C34—H34C	109.5
C17—C16—C15	117.9 (3)	H34B—C34—H34C	109.5
C17—C16—H16	121.1	C36—C35—N8	129.5
C15—C16—H16	121.1	C36—C35—C40	121.0
C16—C17—C18	122.0 (3)	N8—C35—C40	109.5
C16—C17—H17	119.0	C37—C36—C35	117.0
C18—C17—H17	119.0	C37—C36—H36	121.5
C19—C18—C17	121.2 (3)	C35—C36—H36	121.5
C19—C18—H18	119.4	C36—C37—C38	122.1
C17—C18—H18	119.4	C36—C37—H37	118.9
C18—C19—C20	116.5 (3)	C38—C37—H37	118.9
C18—C19—H19	121.8	C39—C38—C37	121.3
C20—C19—H19	121.8	C39—C38—H38	119.4
C19—C20—N3	131.9 (2)	C37—C38—H38	119.4
C19—C20—C15	123.0 (3)	C38—C39—C40	116.8
N3—C20—C15	105.1 (2)	C38—C39—H39	121.6
C31—O2—C30	110.7 (2)	C40—C39—H39	121.6
C27—S3—C28	98.26 (19)	N7—C40—C39	132.2
C27—N5—C26	104.2	N7—C40—C35	105.9
C27—N6—C21	106.1	C39—C40—C35	121.8
C27—N6—C29	129.48 (13)	C33A—S4A—C34A	99.7
C21—N6—C29	124.46 (13)	C40A—N7A—C33A	105.9
N6—C21—C22	131.6	C40A—N7A—C32A	125.9 (5)
N6—C21—C26	105.4	C33A—N7A—C32A	127.7 (5)
C22—C21—C26	123.0	C33A—N8A—C35A	103.7
C23—C22—C21	116.4	N8A—C33A—N7A	115.1
C23—C22—H22	121.8	N8A—C33A—S4A	125.9
C21—C22—H22	121.8	N7A—C33A—S4A	119.0
C22—C23—C24	121.4	S4A—C34A—H34D	109.5
C22—C23—H23	119.3	S4A—C34A—H34E	109.5
C24—C23—H23	119.3	H34D—C34A—H34E	109.5
C25—C24—C23	122.0	S4A—C34A—H34F	109.5
C25—C24—H24	119.0	H34D—C34A—H34F	109.5
C23—C24—H24	119.0	H34E—C34A—H34F	109.5
C24—C25—C26	117.4	C36A—C35A—N8A	129.5
C24—C25—H25	121.3	C36A—C35A—C40A	121.0
C26—C25—H25	121.3	N8A—C35A—C40A	109.5
C25—C26—N5	129.7	C37A—C36A—C35A	117.0
C25—C26—C21	119.9	C37A—C36A—H36A	121.5
N5—C26—C21	110.3	C35A—C36A—H36A	121.5
N5—C27—N6	114.0	C36A—C37A—C38A	122.1
N5—C27—S3	126.5	C36A—C37A—H37A	118.9
N6—C27—S3	119.5	C38A—C37A—H37A	118.9
S3—C28—H28A	109.5	C39A—C38A—C37A	121.3
S3—C28—H28B	109.5	C39A—C38A—H38A	119.4
H28A—C28—H28B	109.5	C37A—C38A—H38A	119.4
S3—C28—H28C	109.5	C38A—C39A—C40A	116.8
H28A—C28—H28C	109.5	C38A—C39A—H39A	121.6
H28B—C28—H28C	109.5	C40A—C39A—H39A	121.6
C27A—S3A—C28A	98.3 (8)	N7A—C40A—C39A	132.2
C27A—N5A—C26A	104.2	N7A—C40A—C35A	105.9
C29—N6A—C27A	125.0 (3)	C39A—C40A—C35A	121.8
			
C7—N2—C1—C2	−177.5 (3)	N6A—C21A—C22A—C23A	178.1
C9—N2—C1—C2	0.5 (4)	C26A—C21A—C22A—C23A	0.3
C7—N2—C1—C6	1.4 (3)	C21A—C22A—C23A—C24A	0.2
C9—N2—C1—C6	179.4 (2)	C22A—C23A—C24A—C25A	−0.3
N2—C1—C2—C3	178.4 (3)	C23A—C24A—C25A—C26A	−0.2
C6—C1—C2—C3	−0.3 (4)	C24A—C25A—C26A—N5A	−177.3
C1—C2—C3—C4	0.4 (4)	C24A—C25A—C26A—C21A	0.7
C2—C3—C4—C5	0.0 (4)	C27A—N5A—C26A—C25A	178.0
C3—C4—C5—C6	−0.4 (4)	C27A—N5A—C26A—C21A	−0.1
C4—C5—C6—C1	0.4 (4)	N6A—C21A—C26A—C25A	−179.0
C4—C5—C6—N1	−177.2 (3)	C22A—C21A—C26A—C25A	−0.8
N2—C1—C6—C5	−179.1 (2)	N6A—C21A—C26A—N5A	−0.7
C2—C1—C6—C5	−0.1 (4)	C22A—C21A—C26A—N5A	177.6
N2—C1—C6—N1	−1.1 (3)	C26A—N5A—C27A—N6A	0.9
C2—C1—C6—N1	177.9 (2)	C26A—N5A—C27A—S3A	−178.4
C7—N1—C6—C5	178.1 (3)	C29—N6A—C27A—N5A	167.2 (9)
C7—N1—C6—C1	0.3 (3)	C21A—N6A—C27A—N5A	−1.3
C6—N1—C7—N2	0.7 (3)	C29—N6A—C27A—S3A	−13.5 (9)
C6—N1—C7—S1	−178.45 (19)	C21A—N6A—C27A—S3A	178.0
C1—N2—C7—N1	−1.4 (3)	C28A—S3A—C27A—N5A	−25.4 (8)
C9—N2—C7—N1	−179.3 (2)	C28A—S3A—C27A—N6A	155.3 (8)
C1—N2—C7—S1	177.80 (17)	C27A—N6A—C29—C30	112.2 (6)
C9—N2—C7—S1	−0.1 (3)	C21A—N6A—C29—C30	−81.8 (5)
C8—S1—C7—N1	−1.8 (3)	C27—N6—C29—C30	105.3 (3)
C8—S1—C7—N2	179.1 (2)	C21—N6—C29—C30	−73.6 (3)
C7—N2—C9—C10	99.1 (3)	C31—O2—C30—C29	−169.6 (2)
C1—N2—C9—C10	−78.4 (3)	N6A—C29—C30—O2	−61.3 (4)
C11—O1—C10—C9	−180.0 (2)	N6—C29—C30—O2	−68.0 (3)
N2—C9—C10—O1	−179.5 (2)	C30—O2—C31—C32	−171.2 (3)
C10—O1—C11—C12	179.9 (2)	C30—O2—C31—C32A	166.8 (5)
C13—N3—C12—C11	103.6 (3)	O2—C31—C32—N7	−160.5 (2)
C20—N3—C12—C11	−76.4 (3)	O2—C31—C32A—N7A	175.8 (6)
O1—C11—C12—N3	−60.0 (3)	C31—C32—N7—C40	80.5 (3)
C15—N4—C13—N3	0.8 (3)	C31—C32—N7—C33	−106.9 (3)
C15—N4—C13—S2	−179.9 (2)	C35—N8—C33—N7	0.9
C20—N3—C13—N4	−0.6 (3)	C35—N8—C33—S4	−178.6
C12—N3—C13—N4	179.4 (2)	C40—N7—C33—N8	−1.4
C20—N3—C13—S2	−179.87 (19)	C32—N7—C33—N8	−175.1 (3)
C12—N3—C13—S2	0.1 (4)	C40—N7—C33—S4	178.2
C14—S2—C13—N4	−23.2 (3)	C32—N7—C33—S4	4.5 (3)
C14—S2—C13—N3	156.0 (3)	C34—S4—C33—N8	−1.8
C13—N4—C15—C16	178.5 (3)	C34—S4—C33—N7	178.7
C13—N4—C15—C20	−0.8 (3)	C33—N8—C35—C36	177.9
N4—C15—C16—C17	−178.7 (3)	C33—N8—C35—C40	−0.1
C20—C15—C16—C17	0.5 (4)	N8—C35—C36—C37	−177.3
C15—C16—C17—C18	−1.6 (5)	C40—C35—C36—C37	0.4
C16—C17—C18—C19	1.4 (6)	C35—C36—C37—C38	0.3
C17—C18—C19—C20	−0.1 (5)	C36—C37—C38—C39	−1.0
C18—C19—C20—N3	178.7 (3)	C37—C38—C39—C40	0.9
C18—C19—C20—C15	−1.0 (4)	C33—N7—C40—C39	−176.9
C13—N3—C20—C19	−179.6 (3)	C32—N7—C40—C39	−2.8 (3)
C12—N3—C20—C19	0.4 (4)	C33—N7—C40—C35	1.1
C13—N3—C20—C15	0.0 (3)	C32—N7—C40—C35	175.2 (3)
C12—N3—C20—C15	−180.0 (2)	C38—C39—C40—N7	177.7
C16—C15—C20—C19	0.8 (4)	C38—C39—C40—C35	−0.1
N4—C15—C20—C19	−179.8 (2)	C36—C35—C40—N7	−178.8
C16—C15—C20—N3	−178.9 (2)	N8—C35—C40—N7	−0.7
N4—C15—C20—N3	0.4 (3)	C36—C35—C40—C39	−0.6
C27—N6—C21—C22	−176.9	N8—C35—C40—C39	177.6
C29—N6—C21—C22	2.2 (2)	C31—C32A—N7A—C40A	−75.9 (9)
C27—N6—C21—C26	1.1	C31—C32A—N7A—C33A	94.9 (7)
C29—N6—C21—C26	−179.8 (2)	C35A—N8A—C33A—N7A	0.9
N6—C21—C22—C23	178.1	C35A—N8A—C33A—S4A	−178.6
C26—C21—C22—C23	0.3	C40A—N7A—C33A—N8A	−1.4
C21—C22—C23—C24	0.2	C32A—N7A—C33A—N8A	−173.6 (8)
C22—C23—C24—C25	−0.3	C40A—N7A—C33A—S4A	178.2
C23—C24—C25—C26	−0.2	C32A—N7A—C33A—S4A	6.0 (8)
C24—C25—C26—N5	−177.3	C34A—S4A—C33A—N8A	−1.8
C24—C25—C26—C21	0.7	C34A—S4A—C33A—N7A	178.7
C27—N5—C26—C25	178.0	C33A—N8A—C35A—C36A	177.9
C27—N5—C26—C21	−0.1	C33A—N8A—C35A—C40A	−0.1
N6—C21—C26—C25	−179.0	N8A—C35A—C36A—C37A	−177.3
C22—C21—C26—C25	−0.8	C40A—C35A—C36A—C37A	0.4
N6—C21—C26—N5	−0.7	C35A—C36A—C37A—C38A	0.3
C22—C21—C26—N5	177.6	C36A—C37A—C38A—C39A	−1.0
C26—N5—C27—N6	0.9	C37A—C38A—C39A—C40A	0.9
C26—N5—C27—S3	−178.4	C33A—N7A—C40A—C39A	−176.9
C21—N6—C27—N5	−1.3	C32A—N7A—C40A—C39A	−4.4 (8)
C29—N6—C27—N5	179.6 (2)	C33A—N7A—C40A—C35A	1.1
C21—N6—C27—S3	178.0	C32A—N7A—C40A—C35A	173.6 (8)
C29—N6—C27—S3	−1.0 (2)	C38A—C39A—C40A—N7A	177.7
C28—S3—C27—N5	−23.16 (19)	C38A—C39A—C40A—C35A	−0.1
C28—S3—C27—N6	157.56 (19)	C36A—C35A—C40A—N7A	−178.8
C29—N6A—C21A—C22A	15.0 (9)	N8A—C35A—C40A—N7A	−0.7
C27A—N6A—C21A—C22A	−176.9	C36A—C35A—C40A—C39A	−0.6
C29—N6A—C21A—C26A	−166.9 (9)	N8A—C35A—C40A—C39A	177.6
C27A—N6A—C21A—C26A	1.1		

### 1,1'-[Oxybis(ethane-2,1-diyl)]bis(2-methylsulfanyl-1H-benzo[d]imidazole) Hydrogen-bond geometry (Å, º)

*Cg*1, *Cg*5 and *Cg*11 are the centroids of the N5/C26/C21/N6/C27, the C21-C26 and the C1–C6 rings, respectively.

**Table:**

*D*—H···*A*	*D*—H	H···*A*	*D*···*A*	*D*—H···*A*
C2—H2···*Cg*5^i^	0.95	2.71	3.583 (3)	153
C11—H11*B*···*Cg*11^ii^	0.99	2.74	3.423 (3)	126
C29—H29*B*···*Cg*1^iii^	0.99	2.70	3.489 (3)	137
C34—H34*B*···N4^iv^	0.98	2.50	3.398 (3)	153

Symmetry codes: (i) −*x*+1, −*y*+1, −*z*+1; (ii) −*x*, −*y*+1, −*z*+1; (iii) −*x*+1, −*y*, −*z*; (iv) −*x*+1, −*y*+2, −*z*+1.
